# An exclusive human milk-based diet in extremely premature infants reduces the probability of remaining on total parenteral nutrition: a reanalysis of the data

**DOI:** 10.1186/1756-0500-5-188

**Published:** 2012-04-25

**Authors:** Heli Ghandehari, Martin L Lee, David J Rechtman

**Affiliations:** 1 UCLA School of Public Health, 405 Hilgard Avenue, Los Angeles, CA 90024, USA; 2 Prolacta Bioscience and UCLA School of Public Health, 605 E. Huntington Drive, Monrovia, CA 91016, USA; 3 Prolacta Bioscience, 605 E. Huntington Drive, Monrovia, CA 91016, USA

**Keywords:** Premature neonates, Human milk nutrition, Total parenteral nutrition, Counting process, Proportional hazards model

## Abstract

**Background:**

We have previously shown that an exclusively human milk-based diet is beneficial for extremely premature infants who are at risk for necrotizing enterocolitis (NEC). However, no significant difference in the other primary study endpoint, the length of time on total parenteral nutrition (TPN), was found. The current analysis re-evaluates these data from a different statistical perspective considering the probability or likelihood of needing TPN on any given day rather than the number of days on TPN. This study consisted of 207 premature infants randomized into three groups: one group receiving a control diet of human milk, formula and bovine-based fortifier (“control diet”), and the other two groups receiving only human milk and human milk-based fortifier starting at different times in the enteral feeding process (at feeding volumes of 40 or 100 mL/kg/day; “HM40” and “HM100”, respectively). The counting process Cox proportional hazards survival model was used to determine the likelihood of needing TPN in each group.

**Results:**

The two groups on the completely human-based diet had an 11-14 % reduction in the likelihood of needing nutrition via TPN when compared to infants on the control diet (p = 0.0001 and p = 0.001, respectively for the HM40 and HM100 groups, respectively). This was even more pronounced if the initial period of TPN was excluded (p < 0.0001 for both the HM40 and HM100 groups).

**Conclusions:**

A completely human milk-based diet significantly reduces the likelihood of TPN use for extremely premature infants when compared to a diet including cow-based products. This likelihood may be reduced even further when the human milk fortifier is initiated earlier in the feeding process.

**Trial registration:**

This study was registered at http://www.clinicaltrials.gov reg. # NCT00506584

## Background

Necrotizing enterocolitis (NEC) is a disease affecting premature infants within the first several weeks of life. It is characterized by inflammation of the gut and often presents symptoms of abdominal distension, bilious vomiting and bloody stools [1,2]. While only 1-5 % of admissions to the neonatal intensive care unit (NICU) are diagnosed with NEC, the incidence of NEC can be as high as 16 % in infant populations weighing less than 1,500 grams [2]. Despite substantial research efforts, the pathogenesis of NEC is not well understood and there is currently no effective prevention of this disease [2].

While the cause of NEC remains unknown, the risk of the disease appears to increase for infants who receive preterm formula [2]. In fact, a lower incidence of NEC and late-onset sepsis has been reported for premature infants who are fed their mother’s breast milk [3]. Unfortunately, not all mothers who give birth to premature infants are able to supply sufficient amounts of breast milk [4]. As a result, there has been extensive research regarding the use of donor human milk as an alternative to preterm formula [5]. A review of this research found a significantly lower incidence of NEC in infants who were given donor human milk instead of formula [6].

There is often reluctance, particularly in the smallest and sickest infants, to begin substantial enteral feedings during the first few days of life. Once enteral feedings are begun, they are usually increased slowly and total parenteral nutrition (TPN) is decreased accordingly. Infants who develop NEC are also treated with total parenteral nutrition to intravenously supply the necessary salts, glucose, amino acids, lipids and vitamins while the patient undergoes bowel rest.

In our previously published study of premature infants weighing less than 1250 grams at birth [1], we demonstrated that a diet consisting of only human milk (maternal breast milk, donor human milk and a human milk-based fortifier, ProlactPlus H [2] MF®) could reduce the incidence of both medical and surgical NEC when compared to a control diet (maternal breast milk fortified with cow milk-based fortifier combined with the use of preterm formula when maternal breast milk was unavailable). We were not able to demonstrate an effect on the length of time the infant was on TPN using a standard time-to-event analysis (Kaplan-Meier methodology). This finding is documented in the original paper, but it is worth noting that there was a difference of only two days in the median length of time on TPN (p = 0.71). However, that analysis treated each day of TPN as the same as any other in computing the time on TPN. In retrospect, it became clear that this was not necessarily true. For example, an infant who received TPN for 20 days in one continuous period was not necessarily equivalent to an infant who initially received TPN for 10 days, successfully achieved full enteral feedings, but then had to return to TPN treatment for an additional 10 days because of gastrointestinal difficulties. Instead, what may matter most is the actual likelihood or probability of needing TPN on any one particular day of life, since the treating physician is primarily concerned about avoiding the use of TPN at any point in the infant’s care. However, it is important to emphasize that the initial course of TPN cannot be affected by whether the infant is placed on 100 % human milk, particularly the human milk-based fortification because this is not typically initiated until later in the initial enteral feeding regimen.

The current analysis aims to reevaluate the TPN data from the Sullivan et al study [1] in order to compare the likelihood of needing supplemental TPN on any given day in premature infants who were on an exclusively human milk-based diet compared to those who received a control diet that included formula and other cow milk-based products.

## Materials and methods

Data were used from a randomized clinical trial conducted in 12 neonatal intensive care units, 11 in the United States and 1 in Austria from mid-2007 to late-2008 [1]. In brief, this study enrolled 207 premature infants who met the following eligibility criteria: birth weight between 500 and 1,250 grams, a reasonable expectation of survival for the duration of the trial, start of TPN by 48 hours of life, ability to begin enteral feedings by the 21^st^ day of life, and an intention for the infant to receive at least some of his/her own mother’s milk. Informed consent was obtained from the parents or guardians of every study subject. The individual IRB/EC approval from each of the 13 study sites was obtained prior to the initiation of the study at each center. (These included the Institutional Review Boards of the University of Florida; Schneider Children’s Hospital; University of California, San Diego Medical Center; Rush University Medical Center; University of Utah Medical Center; University of Texas, San Antonio Health Science Center; Baylor College of Medicine; Duke University Medical Center; University of Rochester/Golisano Children’s Hospital at Strong; Yale University School of Medicine; Children’s Hospital of Oakland and Research Center; Johns Hopkins School of Medicine; and the Ethical Committee of the Innsbruck Medical University).

The infants were randomized into one of three arms, and randomization was stratified by birth weight (500-750 g, 751-1000 g or 1001-1250 g) and size at birth (appropriate- or small-for-gestational-age; AGA and SGA, respectively). The first group of infants (BOV, n = 69) received their mother’s milk with a cow’s milk-based fortifier initiated when nutrition volume reached 100 mL/kg/day and pre-term formula when mother’s milk was unavailable or insufficient. The second group of infants (HM100, n = 67) received their mother’s milk with a human milk-based fortifier initiated when nutrition volume reached 100 mL/kg/day and donor breast milk was supplied as needed to supplement mother’s milk. The third group (HM40, n = 71) had a similar feeding profile to the HM100 group with the exception that the human milk-based fortifier was initiated when nutrition volume reached 40 mL/kg/day. Clinicians were not blinded to the randomized study group because of difficulties in disguising the actual nutrition.

Information was collected regarding the subjects’ demographic and clinical characteristics, including gender, race, birth weight, gestational age, size at birth (AGA vs. SGA), Apgar score at five minutes, administration of antenatal steroids and need for mechanical ventilation at study entry. Daily records were also kept regarding the amount and type of feed received by the infants.

Statistical analysis for the current evaluation was performed using SAS 9.1.3 (SAS Institute, Inc, Research Triangle Park, NC, 2005). The Cox proportional hazards (PH) model was used to determine the likelihood of needing TPN associated with each group. This statistical model allows for the use of right-censored data in the determination of the risk of a particular dichotomous outcome, which in our case is whether an infant required TPN on any given study day. Important clinical covariates for the model included number of days before the first enteral feed (PRETX), initial number of consecutive days on TPN (TPNOFF), and the amount of own mother’s breast milk received per kilogram of infant’s body weight per day (BM), while evaluating the differences in nutritional groups (TX). Note that the occurrence of NEC is not included in this model because of the relatively low incidence of this outcome in the study (19 cases out of 207 or 9.2 %), and the previous demonstration from these data that total TPN days did not differ between NEC and non-NEC infants. However, a subsequent analysis of the model demonstrated that this variable did not affect the model or the outcomes. The hazard or risk function associated with the Cox PH model is exponential in nature and can be described as follows:

$$\text{h}\left(\text{t}\right)={\text{h}}_{0}\left(\text{t}\right)\text{exp}\{\beta \text{TX}+\gamma_{1}\text{PRETX}+\gamma_{2}\text{TNOFF}+\gamma_{3}\text{BM}\}$$

Where β, γ_1,_ γ_2,_ and γ_3_ are coefficients in the model and h_0_(t) is the baseline risk of TPN. In other words, this statistical model allows for the comparison of the study groups while incorporating other potentially important predictors of TPN usage.

**Figure 1:**
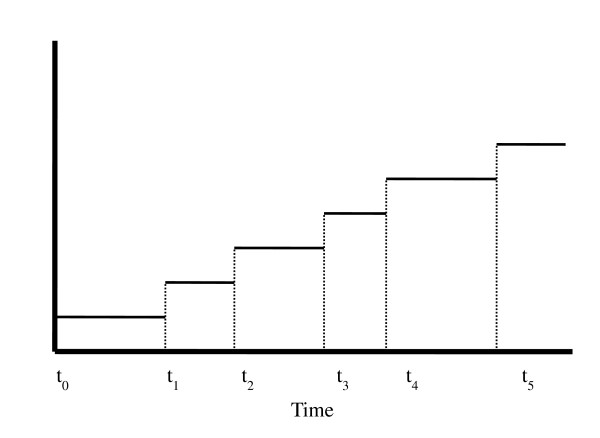
**An example of how event data was compiled using counting process theory.** The graph demonstrates the event times of a single subject; each subsequent event adds to the number of days that the subject has been on TPN. The Cox PH model treats each interval as an independent observation, thus allowing for the occurrence of multiple events.

Many of the infants in the trial required multiple days on TPN, some after having achieved full enteral feeding. The Cox PH survival model is useful because it can take into account the occurrence of multiple events throughout the study period. We applied a statistical method known as counting process theory in order to account for those subjects with more than one time interval in the set of intervals “at risk” for continued TPN usage [7]. Thus, this theory allows for several time-to-event intervals to be considered for a single subject (See Figure 1). The resulting series of events are considered independent despite the fact that several outcomes originate from the same subject. However, this is plausible because the reason for being on TPN at different periods of time for the same infant may vary. Our analysis thereby allows for the calculation of the overall probability or likelihood of needing TPN on any particular day by consolidating the total amount of time the subject spent in this state. However, as noted, this analysis has been simplified by assuming that an interval on TPN is independent (in length) of any other interval on TPN for the same individual. Examination of the data from the current study appears to support this assumption.

## Results

Table 1 provides the parameter estimates associated with the counting process Cox PH model. Compared to infants on BOV diet, those on the HM100 diet had an 11 % reduction in the likelihood of requiring parenteral nutrition (p = 0.001). Those on the HM40 diet had a 14 % reduction in this likelihood compared to the control group (p = 0.0001). There were no significant differences between the HM40 and HM100 groups.

**Table 1:** Parameter estimates and hazard ratios for Cox PH model

**Variable**	**Parameter Estimate**	**Standard Error**	**Hazard Ratio**	**95 % Confidence Interval for Hazard Ratio**	**p-value**
HM100	−0.122	0.038	0.885	0.81, 0.96	0.001
HM40	−0.150	0.039	0.860	0.78, 0.94	0.0001

HM100 = Human milk-based fortifier added once feeding volume = 100 mL/(kg*day).

HM40 = Human milk-based fortifier added once feeding volume = 40 mL/(kg*day).

p-value refers to the comparison to the BOV (control group).

Because the initial period of TPN, which might be unaffected by the type of nutrition used, could have influenced these results, we repeated these analyses without including that period. For this sub-analysis, the reductions in likelihood compared to the BOV group were 24 % and 34 % for the HM100 and HM40 groups, respectively (p < 0.0001). Thus, the effect of 100 % human-milk based nutrition was not mitigated by the elimination of the initial period of TPN from the analysis.

We also evaluated only those infants that did not develop NEC (n = 188) during the course of the trial. Overall, the human milk-based diets together showed a 7 % reduction in the likelihood of requiring TPN (p = 0.06), indicating that the beneficial effect of the enteral diet persisted even in this subsample without NEC which can prolong the need for TPN.

It is also important to note that there were no significant safety issues seen with infants receiving any of these diets.

## Discussion

The original study from which these data were drawn was not able to show that a 100 % human milk based diet could reduce the length of time an extremely premature infant required TPN. Indeed, the study was powered to demonstrate a 7 day median reduction in TPN duration and found only a 2 day difference, which was not statistically significant. However, that analysis overlooked a key point, i.e., that all days of TPN treatment do not carry the same clinical significance. Inevitably, most extremely premature infants begin their nutritional life receiving TPN and the ability to wean them off this and onto enteral nutrition is not necessarily a function of the enteral feeding itself, but may also be a function of the infant’s clinical condition. However, once complete enteral nutrition is achieved, a return to TPN is likely due to the lack of tolerance of enteral feeding. As a result, an analysis of how long overall TPN is needed may miss the benefits associated with an exclusively human milk-based diet. In our current analysis, we have considered what we believe to be a more reasonable statistical approach that acknowledges this fact by evaluating the likelihood of requiring TPN on any given day. This type of analysis “rewards” an approach that would prevent a patient from returning to TPN on at least one occasion and decreases the importance of the duration of the initial course of TPN, which may reflect illness severity and/or unit based feeding practices. Our finding that the effect of 100% human milk-based nutrition on TPN use is even more substantial after elimination of the initial course of TPN from the analysis further emphasizes this point. Therefore, the current analysis provides support to the notion that a 100 % human milk-based diet can reduce the need for TPN by demonstrating that the likelihood of TPN usage on a given day of life is reduced. We believe that this is one of the key goals of a proper nutritional regimen. This finding persisted even after controlling for increased incidence and severity of NEC in the control group suggesting that this effect improves overall feeding tolerance. This result, coupled with our previous report of a significantly reduced risk of medical and surgical NEC, provides further impetus for the adoption of a 100 % human milk-based diet in the NICU for infants under 1250 grams birth weight. The finding that a more aggressive feeding approach by introducing fortification at a feeding volume of 40 mL/kg/day actually improved feeding tolerance, as measured by the greater reduction in risk of requiring TPN, is especially intriguing but needs to be confirmed by further studies.

We note that this study has certain limitations including the lack of information on why enteral feedings were interrupted and TPN restarted and the relatively small sample size which may have affected the ability to detect differences in actual TPN days.

## Conclusions

This secondary analysis of prospectively collected data shows that a 100 % human milk-based diet significantly reduces the likelihood of needing nutrition via TPN for extremely premature infants when compared to a standard diet of human milk supplemented with cow’s milk-based fortifier and pre-term formula when needed. Furthermore, initiating the human milk-based fortifier earlier may result in a further reduction in the likelihood of TPN use in these infants.

## Abbreviations

AGA: Appropriate for gestational age; BM: Volume of breast milk received per kilogram infant body weight; BOV: Control group; EC: Ethical committee; HM40: Human milk group fortification started at 40 mL per kg per day; HM100: Human milk group fortification started at 100 mL per kg per day; IRB: Institutional review board; NEC: necrotizing enterocolitis; NICU: Neonatal intensive care unit; PH: Proportional hazards; PRETX: Number of days before first enteral feed; SGA: Small for gestational age; TPN: Total parenteral nutrition; TPNOFF: Initial number of consecutive days on TPN.

## Competing interests

Support for the study upon which this paper is based was provided by Prolacta Bioscience. Drs. Lee and Rechtman are employees of Prolacta Bioscience. All members of the H2MF Study Group received study-related support from Prolacta Bioscience except for Dr. Meier who was not a clinical investigator. Ms. Ghandehari received no financial support for her work, which originated as a Master's degree thesis under the direction of Dr. Lee.

## Authors’ contributions

HG helped to develop the concept and performed the basic analyses. ML was involved in the design of the original study and the analysis of the study data. DR was involved in the design of the original study and the writing of the manuscript. The H2 MF study group performed the original study and collected the data upon which this manuscript is based. All authors read and approved the final manuscript.

## Author information

The H2MF Study group is comprised of the authors and: Sandra Sullivan MD, Pediatrics, University of Florida, Gainesville, FL, USA; Richard J. Schanler MD, Pediatrics, Schneider Children’s Hospital at North Shore, Manhasset, NY, USA and Albert Einstein College of Medicine, Bronx, NY, USA; Jae H. Kim MD PhD, Pediatrics, University of California, San Diego Medical Center, San Diego, CA, USA; Aloka L. Patel MD, Paula Meier PhD, Pediatrics, Rush University Medical Center, Chicago, IL, USA; Rudolf Trawöger MD, Ursula Kiechl-Kohlendorfer MD, Pediatrics, Innsbruck Medical University, Innsbruck, Austria; Gary M. Chan MD, Pediatrics, University of Utah Medical Center, Salt Lake City, UT, USA; Cynthia L. Blanco MD, Pediatrics, University of Texas Health Science Center, San Antonio, TX, USA; Steven Abrams MD, Pediatrics, Baylor College of Medicine, Houston, TX, USA; C. Michael Cotten MD MHS, Pediatrics, Duke University Medical Center, Durham, NC, USA; Nirupama Laroia MD, Pediatrics, University of Rochester, Golisano Children’s Hospital at Strong, Rochester, NY, USA; Richard A. Ehrenkranz MD, Pediatrics, Yale University School of Medicine, New Haven, CT, USA; Golde Dudell MD, Pediatrics, Children's Hospital and Research Center, Oakland, CA, USA Elizabeth A. Cristofalo MD MPH, Pediatrics, Johns Hopkins School of Medicine, Baltimore, MD, USA; Alan Lucas MD. MRC Child Nutrition Research Center, Institute of Child Health, London, UK.

## Funding

Funding for the primary study upon which the current data analysis is based was provided by Prolacta Bioscience.
